# Molecular characterisation of aromatase inhibitor-resistant advanced breast cancer: the phenotypic effect of *ESR1* mutations

**DOI:** 10.1038/s41416-018-0345-x

**Published:** 2018-12-19

**Authors:** Elena Lopez-Knowles, Alex Pearson, Gene Schuster, Pascal Gellert, Ricardo Ribas, Belinda Yeo, Ros Cutts, Richard Buus, Isaac Garcia-Murillas, Ben Haynes, Lesley-Ann Martin, Ian Smith, Nick Turner, Mitch Dowsett

**Affiliations:** 1 The Breast Cancer Now Toby Robins Research Centre at the Institute of Cancer Research, London, UK; 2 Ralph Lauren Centre for Breast Cancer Research, Royal Marsden Hospital, London, UK; 3 Breast Unit, Royal Marsden Hospital, London, UK; 4 Olivia Newton-John Cancer Research Institute, Melbourne, VIC Australia

**Keywords:** Breast cancer, Molecular medicine

## Abstract

**Background:**

Several thousand breast cancer patients develop resistance to aromatase inhibitors (AIs) each year in the UK. Rational treatment requires an improved molecular characterisation of resistant disease.

**Materials and methods:**

The mutational landscape of 198 regions in 16 key breast cancer genes and RNA expression of 209 genes covering key pathways was evaluated in paired biopsies before AI treatment and at progression on AI from 48 patients. Validity of findings was assessed in another five ESR1-mutated tumours progressing on AI.

**Results:**

Eighty-nine mutations were identified in 41 matched pairs (*PIK3CA* in 27%; *CDH1* in 20%). *ESR1* (*n* = 5), *ERBB2* (*n* = 1) and *MAP2K4* (*n* = 1) had mutations in the secondary sample only. There was very high heterogeneity in gene expression between AI-resistant tumours with few patterns apparent. However, in the *ESR1*-mutated AI-resistant tumours, expression of four classical oestrogen-regulated genes (ERGs) was sevenfold higher than in *ESR1* wild-type tumours, a finding confirmed in the second set of *ESR1*-mutated tumours. In *ESR1* wild-type AI-resistant tumours ERG expression remained suppressed and was uncoupled from the recovery seen in proliferation.

**Conclusions:**

Major genotypic and phenotypic heterogeneity exists between AI-resistant disease. ESR1 mutations appear to drive oestrogen-regulated processes in resistant tumours.

## Introduction

Aromatase inhibitors (AIs) are the standard of care as first-line treatment for postmenopausal women with oestrogen receptor positive (ER + ) advanced breast cancer (BC).^1^ However, the objective response rate to AIs in the metastatic setting is between 20%–40% and virtually all patients eventually relapse with AI-resistant disease.^2,3^ It is critical to understand the molecular drivers of the resistance to allow rational use of subsequent or concurrent therapy. Several potential mechanisms of resistance have been described including changes in the expression of ER or its coregulators, as well as the *ESR1* mutational status. *ESR1* mutations in the ligand-binding domain of ER lead to constitutive activity in model systems^4^ and have been detected in 15–20% of patients with metastatic ER + endocrine resistance BC^5–10^; up to 40% of patients have been reported to have *ESR1*-mutated circulating tumour (ct) DNA.^11^ Other potential mechanisms of resistance to endocrine therapy include the activation of signalling pathways such as the PI3K/mTOR pathway.^12^

Paired tumour biopsies before and at recurrence or progression on AIs are infrequently available. However, in our previous report of 55 such pairs we found a highly variable immunohistochemical phenotype of several candidate markers between pre-AI and AI-resistant biopsies.^13^ Others^14^ have reported similar observations that indicate that multiple mechanisms of resistance occur to AI. While loss of ER occurred in some cases, others recurrences showed enhanced expression of ER suggesting persistent ER functioning but downstream markers of such functioning were not measured to confirm or refute this. Other biopsy pairs showed loss of PTEN or HER2 gain, which are consistent with experimental studies of resistance to oestrogen deprivation.^15,16^

To further investigate the range of molecular changes that are associated with AI-resistance, we analysed the same sample set^13^ using a targeted NGS panel to identify somatic mutation in 16 key genes and a Nanostring panel of 209 genes to identify changes in gene expression in major signalling pathways. We found that the majority of mutations in the AI-resistant tumour were shared with their paired pre-AI sample, but almost half of the pairs showed at least one private mutation. *ESR1*, *ERBB2* and *MAP2K4* had mutations in the secondary sample only, while there was no systematic difference between the primary and secondary sample for the other analysed genes. The expression of classically oestrogen-dependent genes that are downregulated in almost all AI-treated tumours^17^ supported a significant phenotypic impact of ESR1 mutations providing further evidence for the likely benefit from some therapeutic interventions.

### Materials and methods

#### Patient selection and characteristics

**Fig. 1:**
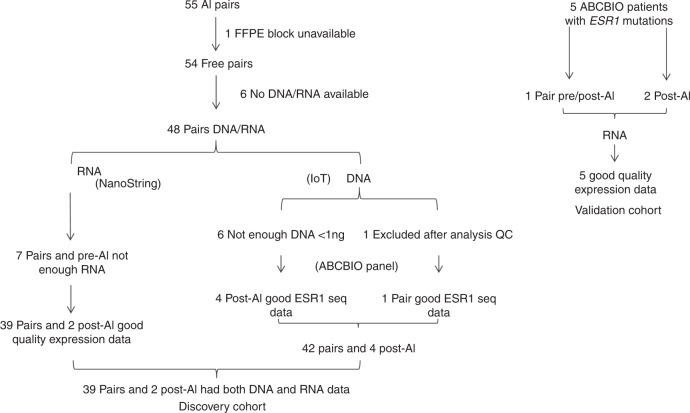
Consort diagram of the 55 AI paired samples (discovery cohort, left) and five ESR1 mutant samples from the ABC-BIO study (validation cohort, right)

Samples used in this study have been described previously.^13^ In brief, 55 patients with ER+ breast cancer from The Royal Marsden Hospital were retrospectively selected if they had relapsed or progressed during AI treatment in the locally advanced or metastatic setting (Discovery cohort, Fig. 1). Patient characteristics and clinical management are summarised in Table 1. 37/48 (77%) of patients received endocrine therapy prior to treatment with an AI, with 31/48 (65%) receiving tamoxifen. 5/48 (10%) patients received both tamoxifen and an AI. Paired tissue blocks, pre and post-AI treatment, from 48 patients were available for DNA and RNA extraction. Of these 48 patients, a total of 21 patients received tamoxifen prior to the pre-AI sample being collected.

**Table 1:** Patient demographics. The clinical characteristics of 48 patients with mutational and/or gene expression data

Clinical characteristics		*n* (%)
Diagnosis		
Age (years)	Mean	54
	Range	27–86
Disease status	EBC	41 (85)
	Locally advanced	5 (10)
	Metastatic	2 (5)
Age at start of AI treatment (years)	Mean	62
	Range	33–88
Pre-AI biopsy		
Site	Primary	30 (62)
	Local recurrence	17 (35)
	Distant recurrence	1 (2)
Disease status	EBC	24 (50)
	Locoregional recurrence	20 (42)
	MBC	4 (8)
AI therapy b/w 1st and 2nd biopsy		
Type	Letrozole	25 (52)
	Anastrozole	21 (44)
	Exemestane	2 (5)
Disease setting for AI therapy	Adj/neoadj	9 (19)
	Local recurrence	25 (52)
	Metastatic	14 (30)
Post-AI biopsy		
Site	Primary	7 (15)
	Local recurrence	26 (54)
	Distant recurrence	15 (31)
Disease status	EBC	3 (6)
	Locoregional recurrence	17 (36)
	MBC	28 (58)
Endocrine therapy prior AI treatment	None	11 (23)
	Tamoxifen	31 (65)
	Tamoxifen + AI	5 (10)
	Grosrelin	1 (2)
Endocrine therapy after PD on AI	AI	31 (65)
	Tamoxifen	7 (15)
	Fulvestrant	5 (10)
HER2 status of either tissues	HER2 positive^a^	7 (15)
	Trastuzumab received	6 (13)
Overall survival^b^ (years)	Median	8.75
	Range	2-33

*EBC* early breast cancer, *MBC* metastatic breast cancer, *AI* aromatase inhibitor, *PD* progressive disease

^a^Either 1st or 2nd tissue sample

^b^Defined as time from first breast cancer diagnosis to death (alive patients censored)

To assess the validity of observations made in the discovery cohort on the phenotype of tumours with *ESR1* mutations, a set of biopsies from 5 patients with recurrent disease already known to have *ESR1* mutations post-AI treatment was obtained from the ABC-BIO study (Validation cohort, Fig. 1). The ABC-BIO study recruits patients at the Royal Marsden Hospital with advanced breast cancer with accessible metastatic deposits for DNA sequencing using the Breast NGS v1.1 probe set including probes to capture ESR1. Biopsies from three other patients in the ABC-BIO study that were known to harbour *ESR1* mutations but had ceased AI treatment for at least 4 weeks prior to biopsy were excluded because of the potential impact on gene expression.

Essential details of molecular analysis are stated below and fully detailed in the supplementary materials.

#### DNA and RNA extraction

Patients had an FFPE tumour biopsy pre- and post-AI treatment. Tissue sections were microdissected and DNA and RNA were co-extracted using the AllPrep DNA/RNA FFPE Kit (Qiagen, Hilden, Germany), with an extended overnight digestion for the DNA extraction being the only modification from the manufacturer’s instructions. Quantification was done using high sensitivity RNA and DNA Qubit assays (Thermo Fisher Scientific, Carlsbad, CA) and on a Bio-Rad QX200 droplet digital PCR (ddPCR) using RNAseP (Thermo Fisher Scientific).^11^ Samples from the validation cohort were also extracted following the same protocol; however, only one of five cases had a pre-AI treatment block available.

#### Ion Torrent (IoT) PGM sequencing

DNA from the discovery cohort was amplified using a custom panel targeting 198 regions within 16 genes. These genes represent the most mutated genes in breast cancer. Five genes (*CDH1*, *GATA3*, *MAP2K4, MAP3K1*, *PTEN*) were covered between 73 and 100%, while for the other 11 genes (*AKT1*, *BRAF*, *ERBB2, ESR1*, *KIT*, *KRAS*, *PIK3CA*, *PIK3R1*, *RUNX1, SF3B1*, *TP53*), amplicons for known hotspot regions were designed, resulting in a 100% coverage, except for *ERBB2* (90%) and *RUNX1* (5%). Libraries were prepared with 10 ng of DNA and sequenced to a median depth of 782X using the Ion Ampliseq Library Kit v2.0 (Thermo Fisher Scientific).

#### MiSeq and NextSeq sequencing

DNA from 5 tumours from the discovery cohort that were unsuccessful with Ion Torrent and 8 from the validation cohort were run on the Miseq or NextSeq (Illumina, San Diego, CA) using the Breast NGS v1.1 probe set. Protocol and analysis details are described in supplementary materials. For the purposes of this report only *ESR1* mutational data was extracted.

#### Mutational validation

Selected *ESR1*, *TP53*, *HER2*, *MAP2K4*, *MAP3K1* and *PIK3CA* mutations were validated by droplet digital PCR (ddPCR) on a QX200 ddPCR system (Bio-Rad, Hercules, CA), with primers (900 nM) and probes (250 nM) and annealing temperatures described in Table S1. Cycling conditions and calculation of mutant concentration were described previously.^11,18^

*PIK3CA* C420R and E418K and *GATA3* K358fs mutations were validated by cycle sequencing.

#### Nanostring gene expression analysis

RNA was run on a NanoString nCounter™ with two custom gene expression panels that comprised of 194 genes in CodeSet 1 and 70 genes in CodeSet 2, according to manufacturer’s guidelines. These were comprised of reference genes, the PAM50 gene set and genes involved in steroid hormone synthesis, ER targets, receptor tyrosine kinases, cell cycle/proliferation, apoptosis, cell signalling, mTOR and APOBEC (Table S2A and S2B). Intrinsic subtypes were identified by NanoString Technologies using a proprietary algorithm. NanoString was performed for39 pairs and 2 post-AI samples from the discovery cohort and 1 pair and 2 post-AI from the validation cohort.

#### Statistical analysis

Statistical tests were performed as indicated using either R v3.2.3 or Graphpad Prism v7. *P*-value < 0.05 was considered statistically significant. Where appropriate paired analyses were performed.

## Results

### Discovery cohort

#### Population

A consort diagram showing the sample availability in the population is provided in Fig. 1. The clinicopathological characteristics of the 48 sample pairs with adequate either DNA and/or RNA data are shown in Table 1. In summary, the first tissue sample (pre-AI) was taken most frequently (62%) from the primary BC or from a local recurrence (35%). At the time of this sample, 50% of patients had early disease, 42% had loco regional relapsed disease and 8% had metastatic BC. The second, post-AI tissue was most frequently (54%) from a site of local recurrence. At the time of the post-AI tissue, 58% of patients had metastatic disease, 36% had loco regional recurrence and for 6% of patients the post-AI tissue represented progression in the primary after neoadjuvant AI.

#### Ion Torrent mutational landscape

**Fig. 2:**
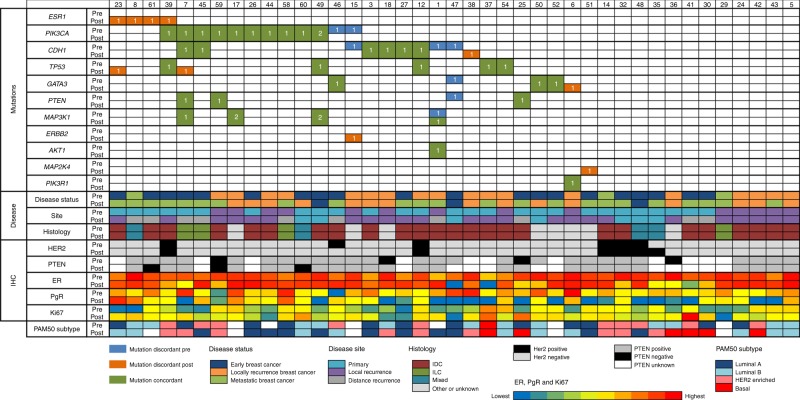
Mutation matrix. All somatic mutations in the coding sequence (CDS) are shown together with IHC expression, clinicopathological parameters and PAM50 subtypes. 1 and 2 indicate the number of mutations identified

Using stringent criteria (see supplementary material), we identified a total of 89 somatic mutations (47 unique genomic positions) among the 41 pairs of sample with adequate DNA and that passed QC, Table S3). The mutations are shown for individual patients in Fig. 2 along with data on PAM50 subtype and previously reported IHC status for ER, PgR, PTEN, Ki67 and HER2 (FISH as necessary). Across all samples, 36 mutations were found in both the primary and secondary samples (shared mutations) whilst 18 mutations were private to one sample of the pair (Fig. S1). For the mutations that were identified in both paired samples, there was no significant difference in variant allele frequency (VAF) between the samples (data not shown). For many pairs we found at least one mutation with high VAF in both samples suggesting a common founding clone. There was no significant difference between the total number of mutations identified on the pre and post samples. The most frequently mutated gene was *PIK3CA* (27%) followed by *CDH1* (20%). Three genes: *ERBB2* (L755S), *MAP2K4* (located at Intron 9-10) and *ESR1* (D538G and E380Q) were mutated exclusively in the post sample and were exclusive of each other. Mutations were validated by ddPCR and cycle sequencing (Table S4) with identified VAFs similar to those found by sequencing, demonstrating high reproducibility of the data. Of the 12 sample pairs with no mutations detected, three were HER2 positive and four had a marked decrease of ER staining in the post-AI sample. Both of these phenotypes might lead to less selective pressure for the acquisition of mutations.

#### *ESR1* mutations

To complement the *ESR1* mutational analysis five further samples from the discovery cohort that were unsuccessful with Ion Torrent were run with an NGS Breast v1.1 panel (Supplementary Materials). This identified one additional *ESR1* mutation in a post-AI sample. This mutation was a previously unreported substitution followed by an insertion at the aa536 hotspot of known mutations (L536indelGV). In all of the five patients with *ESR1* mutations the resistant biopsy was in the metastatic setting (Fig. S2). In one of these cases (patient 23) an intermediate sample taken after 5 years of tamoxifen in the metastatic setting and before AI treatment was available and was found to be *ESR1* wild type.

#### Gene expression

For five genes both IHC and gene expression data (Table S5) were available and for all of these there was a strong significant correlation between the two measurements (Table S6).^19^

**Fig. 3:**
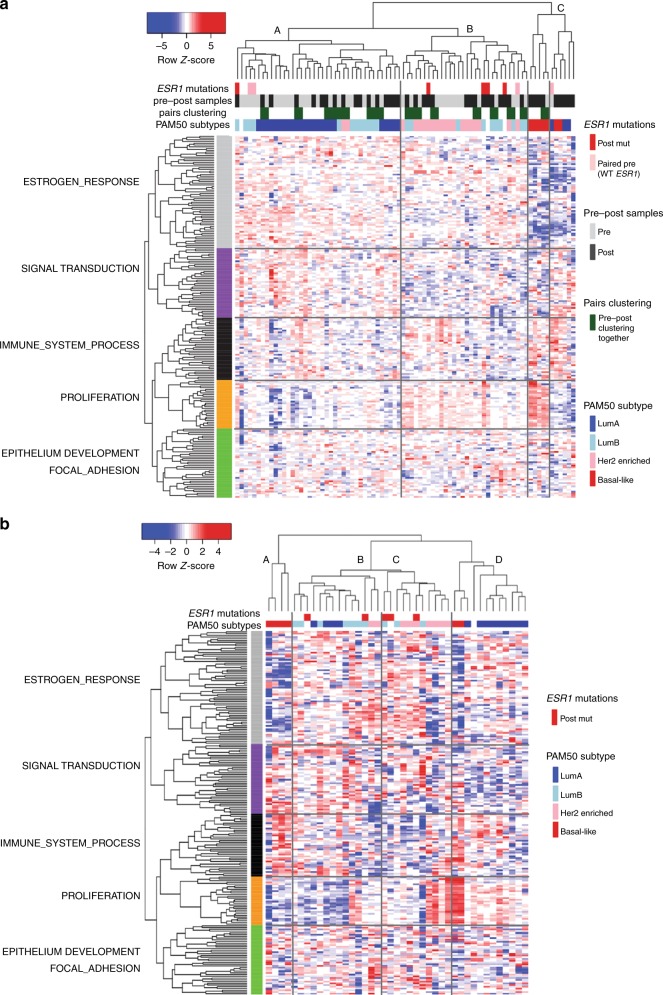
**a** Hierarchical clustering of the 39 sample pairs and two unpaired post samples by gene expression. *ESR1* mutational status, pair pre- and post-AI status (together with pair clustering) and PAM50 subtypes are indicated at the top of the cluster. Five gene (row) clusters are annotated by most significant terms generated from compute overlaps analysis in Broad Institute GSEA website (http://software.broadinstitute.org/gsea/msigdb/annotate.jsp). **b** Hierarchical clustering of the 41 post samples by gene expression. *ESR1* mutational status and PAM50 subtypes are indicated at the top of the cluster. Five gene (row) clusters were taken from clustering used in Fig. 2

Two-way hierarchical clustering of the global gene expression in the pre- and post-AI groups showed 38% (15/39) of pairs clustered together (Fig. 3a). Thirty-six pairs (plus two pre- and two post-AI samples) had PAM50 subtype calculated (Table S7). Only 56% of sample pairs maintained their PAM50 subtype at progression after AI treatment (Table S8). Of particular note only one case was classified as basal-like at baseline but six were classified as basal-like at resistance. Low expression of oestrogen response genes were a consistent feature of this group. The clustering shows some distinct patterns with three major branches labelled A, B and C in Fig. 3a. Branch A consists largely of luminal A and luminal B samples with substantial heterogeneity between them. Branch B consists mainly of HER2-enriched samples and some luminal B. In contrast branch C contains all of the basal-like samples, most of which were unpaired post-treatment samples. The proliferation group of genes appeared to be the dominant feature in clustering the samples most notably into 2 sub-clusters of branch C.

Figure 3b shows 2-way hierarchical clustering of just the AI-resistant samples. While four main clusters can be recognised, the very wide heterogeneity in gene expression in these samples is evident with few groupings due to consistent patterns of expression across the gene set. A small group of tumours with basal-like features (branch A) again segregated from the others based mainly on low expression of oestrogen-regulated genes and high expression of genes in the immune cluster. The central two clusters (B and C) in Fig. 3b differ from the others mainly by their higher expression of oestrogen-regulated genes and contain the ESR1-mutated tumours (see below). The segregation of clusters B and C from one another is then related mainly to proliferation-associated genes. Notably, those with the relatively high proliferation were associated with relatively high signal transduction and immune signalling. The segregation of the cluster classified as HER2-enriched was unexpectedly not dependent on high levels of genes associated with signal transduction but rather on either relatively high proliferation or relatively low expression of immune-related genes.

**Fig. 4:**
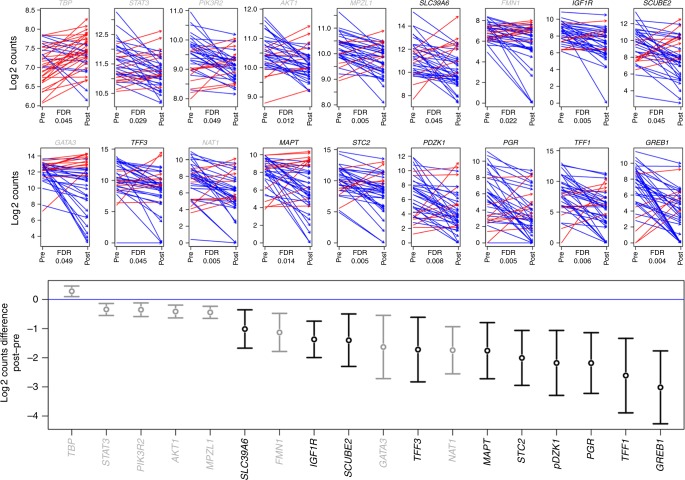
Upper panel, arrow plot of 18 genes that changed significantly pre- and post-AI. Red arrows identify increase of expression in the paired post sample and blue arrows a decrease in expression. FDR values for Student’s *t*-test are shown. Lower panel, box plots of the same 18 genes with mean and 95% confidence interval of log2 difference between paired pre and post samples. Genes coloured in black are ERG genes

Eighteen genes were significantly (FDR 5%) downregulated and one (TBP) was upregulated at progression after AI (Fig. 4). Ten of the 13 most markedly downregulated were known to be subject to regulation by oestrogen signalling. After exclusion of ER negative samples 13/18 genes were significantly differentially expressed. The five genes no longer significantly different were *TFF3, SCUBE2, SLC39A6, TBP, PIK3R2* and *GATA3*. This indicates that suppression of a major axis of oestrogen regulation is maintained despite these tumours demonstrating clinical resistance to AI. Further, expression of *ESR1* and ERa show a strong correlation with the significantly differentially expressed genes (Fig. S3A). The discovery cohort is phenotypically heterogeneous, yet unsupervised clustering of the 18 differentially expressed genes reveals robust downregulation of ERGs in the majority of tumours (Fig. S3B).

Twenty-one patients with paired samples, of which 16 have expression data, had received tamoxifen prior to the pre-AI sample being collected and conceivably this could have impacted on the expression of these 18 differentially regulated genes in the pre-AI sample. However there was no significant difference in gene expression for any of the genes according to prior tamoxifen treatment (Fig. S4). This lack of effect of prior tamoxifen may be due to the drug’s partial agonist activity which is marked in postmenopausal women.^20^

#### ESR1 mutation and gene expression

There was no significant difference in expression of four oestrogen-regulated genes (TFF1, GREB1, PDZK1 and PgR) that we have previously used as markers of oestrogenic signalling,^17^ in the pre-AI samples from the five patients in the discovery cohort that went on to acquire an *ESR1* mutation compared with those that did not (Fig. S5). In four of the five cases it was notable however that oestrogen-regulated gene expression was in the upper range of that in all samples. Expression of the four oestrogen-regulated genes in post-AI samples with *ESR1* mutations was on average more than twofold higher than in *ESR1* wild-type samples for individual genes, and the average expression of these genes in post-AI samples with *ESR1* mutations was more than 6-fold higher than in post-AI samples with wild-type *ESR1* (MannWhitney *P* = 0.006, Fig. S5).

We used the validation cohort to assess the consistency of these observations of a relationship between oestrogen-regulated gene expression and *ESR1* mutations. This cohort consisted of an additional five metastatic samples with previously described *ESR1* mutation in a sample taken after AI treatment increasing the number of *ESR1*-mutated cases with gene expression data to 10. The clinicopathological characteristics of the samples (1 pair and 4 Post-AI samples) are shown in Table S9 and the treatment chronology from diagnosis to death is shown in Fig. S6.

Gene expression of 33 genes was significantly different in the progression sample between *ESR1* wild-type and the ten-mutated tumours (Fig. S7). FOXO3a was the only gene observed to have lower expression in *ESR1* mutant post-AI samples. Using Fisher’s exact test, the remaining 32 genes with higher expression in *ESR1* mutant post-AI samples were significantly enriched for annotations associated with proliferation and most markedly with oestrogen regulation. Five of the genes are part of the 11-gene proliferation signature in PAM50^21 ^(*P* = 0.02, fisher exact test), and 11 are oestrogen-regulated (GSEA Molecular Signature Database Hallmark of Oestrogen Response Early/Late,^22^ *P* = 0.01, fisher exact test). In addition, two of these genes (MELK and BIRC5) are associated with worse outcome or metastasis.^23,24^ After exclusion of ER negative samples, 25/33 genes were significantly differentially expressed, including 8/10 ERGs and the five genes from the PAM50 proliferation signature. The eight genes no longer significantly different were *IL6ST, PGR, FOXO3A, FKBP4, HRAS, KIF2C, CXXC5* and *RPLP0*.

**Fig. 5:**
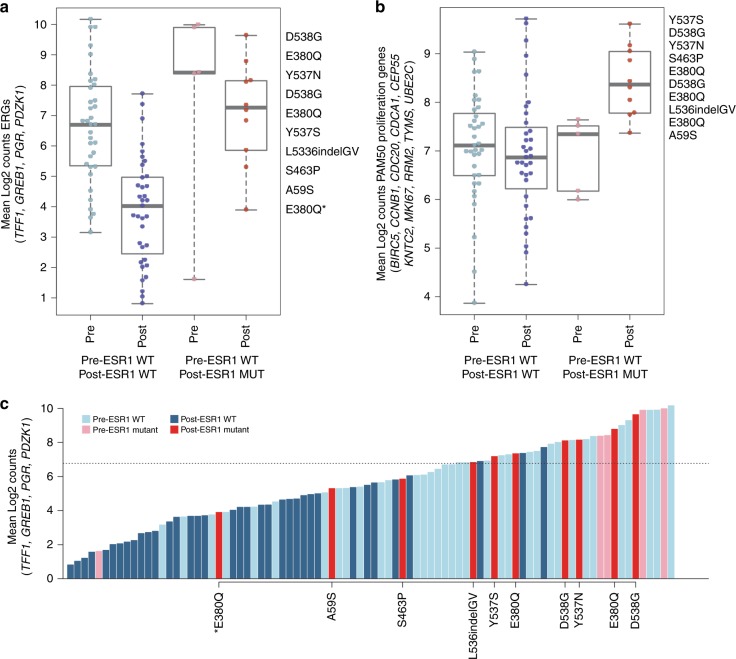
**a** *ESR1* mutations and avERG expression. Box plots of the average expression of TFF1, GREB1, PgR and PDZK1 are shown in the Pre- and Post-AI samples in *ESR1* WT and 13 MUT samples (five from AI study and eight from additional cohort). **b** *ESR1* mutations and PAM50 proliferation gene expression. Box plots of the average expression of the PAM50 proliferation genes are shown in the Pre- and Post-AI samples. **c** Waterfall plot of *ESR1* mutational status and ERG expression. The dashed line represents the mean of all Pre samples. *Indicates a Post-AI *ESR1* mutant sample that is HER2 positive

Figure 5a shows the associations between oestrogen-regulated gene (ERG) expression and *ESR1* mutational status between all ten ESR1-mutated cases and the non-mutated cases according to baseline or post-treatment status. Post-AI samples with *ESR1* mutations had more than sevenfold higher ERG expression than post-AI wild-type samples (MannWhitney *P* = 1.7e–6). Figure 5b shows no significant differences in the PAM50 proliferation genes between the post-treatment samples according to *ESR1* mutation status. A linear scale plot emphasises the magnitude of the difference in ERG expression between post-AI samples with or without *ESR1* mutation (Fig. S8) and the separation in the samples according to ERG expression is particularly clear when shown in a waterfall plot (Fig. 5c). It is notable that the post-AI *ESR1*-mutated tumour with the lowest oestrogen-regulated expression carried an E380Q mutation and was also HER2-positive though this is the only ESR1-mutated sample with HER2 overexpression making the importance of its association with low ERG expression uncertain.

## Discussion

Several thousand women diagnosed with ER + breast cancer recur each year with endocrine resistant disease. The majority are postmenopausal and almost all will have received an AI before or after their recurrence and will require management of their AI-resistant disease. Many potential mechanisms have been reported in model systems but few of these have been confirmed as being associated with AI-resistance in the clinic. To a large degree this is because tissues are difficult to acquire in which to study such associations. The collection of paired pre-AI and AI-resistant tissues assessed here for mutational status and expression levels of BC associated genes although modest in size is therefore an uncommon cohort. Our earlier report revealed very marked heterogeneity between resistant tumours in key IHC biomarkers.^12^ Of note, ER expression was maintained or enhanced in the majority of tumours and was felt to be consistent with a potential for oestrogen signalling in the face of AI to be a driver of resistance, a mechanism that is supported in only a minority of ER + resistant tumours in the current study.

Our data support those from more wide-ranging studies of metastatic breast cancer, in that there was an absence of observed major increases in the acquisition of driver mutations in metastases^10,25,26^ at least among the selected panel of frequently mutated genes assessed. The only gene that differed substantially was *ESR1* in which mutations have been described to be markedly enriched in metastases after AI treatment.^5,7–10^ In this study we identified *ESR1* mutations in 11% of patients, which is at lower end of the reported frequency. This may be due to many of our samples being local recurrences.

*ESR1* mutated recurrent breast cancer has become a focus of attention in the possible development of new agents, such as selective oestrogen receptor degraders but very little has been reported on the phenotype of the *ESR1-*mutated tumours. Evidence from model systems indicates the ligand-independent activity of the hotspot *ESR1* mutations.^4,27–29^ Our clinical data on the significantly higher expression of ERGs when *ESR1* mutations were present, despite the on-going treatment with AI, supports this being valid in clinical tissues. While our observation was made on a relatively small number of samples, it was validated by examination of another cohort from an on-going study of the clinical importance of mutations in metastatic breast cancer. The co-association of the high ERG expression and high proliferation genes in the ESR1-mutated tumours is consistent with the tumour progression being at least partly driven by the mutations. In contrast, the continued suppression of the ERG expression in tumours in which mutations were not detected implies a disconnect between proliferation and oestrogen signalling. Persistent suppression of ERG expression is clearly not a signal for continued anti-tumour effectiveness of the AI: assessment of these genes as a pharmacodynamics marker in this instance would likely be misleading.

We observed small numbers of other mutations that could underpin resistance in individual patients. These included a *MAP2K4* mutation which likely disrupts splicing and potentially leads to not recognising exon 9 by the spliceosome or retaining the intron downstream of exon 9 and the *ERBB2* L755S which has been previously associated with lapatinib resistance^30^ but has also been associated with response to the alternative HER2 tyrosine kinase inhibitor, neratinib.^31^

*PIK3CA* and *TP53* are the most commonly mutated genes in BC with over 30% of patients carrying mutations in either of these genes (IntOgen database^32^). In our study we found that 27% of the patients had mutations in one or both of their samples in *PIK3CA*, but only 15% had a *TP53* mutation (likely due to targeting of *TP53* hotspots in our targeted panel). We also found many patients with a *CDH1* mutation (20%). Loss of CDH1 is a common feature of lobular breast cancer which is almost always ER + . *CDH1* controls the cellular adhesion dynamics^33^ and its loss has been associated with increased cancer invasion.^34^ These features might explain the unusually high frequency in this selection of patients, all of whom relapsed after AI treatment.

There was little consistency other than marked downregulation of ERGs in most patients in recurrent samples. PAM50 subtypes were maintained in > 55% of patients in agreement with the 61% recently described in matched primary and metastatic pairs.^5^ The meaning of the intrinsic subtypes in metastatic disease is however unclear particularly when, as in this study, transcriptional features that underpin the subtyping are impacted by medical therapy.

The most notable feature of the gene expression analyses was the very high degree of heterogeneity between recurrent tumours; this was apparent even within the three or four main clusters identified. This does not necessarily imply that gene expression profiling of recurrent tumours is without value. Rather it supports the need for individualised interpretation of profiles for individual tumours. This is especially so with regard to features such as oestrogen regulation, that might imply the likely benefit or not of alternative targeting of oestrogen signalling, or individual signal transduction pathways that align with particular inhibitors.

Some weaknesses in the current study need to be considered. Many patients had received chemotherapy or tamoxifen prior to the pre-AI sample and then progressed after being treated with an AI. Although prior treatment with tamoxifen might have been expected to impact on gene expression, particularly of known oestrogen-regulated genes, our analyses revealed no significant effect of this prior treatment on the main gene changes noted. Our mutational and transcriptional characterisation was based around features known to be of relevance in breast cancer. An assessment at a more genome-wide level would require a much larger sample set to have confidence in novel observations.

In summary, there is major inter-tumour heterogeneity of genotypic and phenotypic features that may drive resistance to AIs in recurrent breast cancer, requiring highly individualised interpretation of likely dominant pathways in particular cases. Mutational analysis of recurrent disease is of value in identifying targetable abnormalities. Mutations in ESR1 gene are frequently acquired in recurrent disease, having enhanced ERG expression alongside high proliferation-associated genes provides a strong rationale for their targeting with novel agents targeted at the degradation of ligand-independent ER.

## Electronic supplementary material

ai_pairs_Supplementary material_v2.docx

Figure S1

Figure S2

Figure S3

Figure S4

Figure S5

Figure S6

Figure S7

Figure S8

ai_pairs_Supplementary Table 1

ai_pairs_Supplementary Table 2

ai_pairs_Supplementary Table 3

ai_pairs_Supplementary Table 4

ai_pairs_Supplementary Table 5

ai_pairs_Supplementary Table 6

ai_pairs_Supplementary Table 7

ai_pairs_Supplementary Table 8

ai_pairs_Supplementary Table 9
