# Supplementary material for: Molecular characterisation of aromatase inhibitor-resistant advanced breast cancer: the phenotypic effect of ESR1 mutations
Source: Br J Cancer. 2018 Dec 19;120(2):247–55. doi: 10.1038/s41416-018-0345-x (PMC6342946; doi:10.1038/s41416-018-0345-x)
Supplement: Supplementary file 1 — ai_pairs_Supplementary material_v2.docx [file 41416_2018_345_MOESM1_ESM.docx]

**Additional materials and methods**

Ion PGM sequencing

Generated amplicons were combined, primers partially digested, Ion Xpress^TM^ Barcode adapters ligated for tagging and purified with Agencourt AMPure XP magnetic beads (Beckman Coulter, Brea, CA). Libraries were diluted to 7-13pM and templated on the Ion OneTouch2 System (Life Technologies) using Ion PGM Template OT2 200 kit. Templated libraries were then sequenced on the Ion PGM System (Life Technologies) using 500 flows on five 318^TM^ chips.

Reads were aligned by the PGM server with standard settings to the reference genome hg19. On-target coverage was calculated using samtools (v1.2).

BAM files were submitted to IonReporter^TM^ (v4.4) for mutation calling. The following high stringent parameters were chosen: Data Quality Stringency = 12, Downsample To Coverage = 4000, SNP/InDel/MNP (Multiple nucleotide polymorphism) Min Cov Each Strand = 50, SNP/InDel/MNP Min Variant Score = 15, SNP/InDel/MNP Min Coverage = 250, Hotspot Min Variant Score = 6, Hotspot Min Coverage = 150. Transversion to Transition (Ts/Tv) ratio was calculated per sample using vcftools[1] (v0.1.12). Mutations with C>T and G>A transitions with less than 10% were removed using a custom Python script. Mutations were annotated with Tier levels [2] using BedTools[3] (v2.22.1). Annovar[4] (v2015-03-22) was used to annotate with COSMIC[5] (v70) and known germline mutations from the 1000 Genomes Project (1000g2014oct_all) and the NHLBI GO Exome Sequencing Project (ESP) (esp6500siv2). Mutations with any alternative allele frequency in these projects were removed. All mutations called in only one sample of a pair were manually reviewed and included in the analysis if they had a VAF of 1% or more in the other sample.

After mutation calling we detected high transversion to transition (Ts/Tv) ratios for some of the samples, which originate from increased C>T and G>A mutations. This effect is known for FFPE samples in which the hydrolytic de-amination of cytosine results in uracil (or thymine if the cytosine is methylated). Since this effect is random and occurs at different locations in each cell, the resulting variance allele fractions (VAF) of such DNA damages are low [6]. Therefore we filtered C>T and G>A mutations with VAF less than 10% and it dramatically reduced the Ts/Tv ratio for most samples.

Miseq/NextSeq sequencing

The Breast NGS v1.1 panel was used to detect *ESR1* mutations. This panel covers 41 genes either recurrently altered or clinically actionable in metastatic breast cancer. Single nucleotide variants are detected at >5% allele frequency with >99% sensitivity and >98% specificity. Library preparation was performed using the KAPA HyperPlus Kit (Kapa Biosystems, Wilmington, MA, USA) and SeqCap EZ adapters (Roche, NimbleGen, Madison WI, USA), following the manufacturer’s protocol, including dual-SPRI size selection of the libraries (250-450 bp). Following fragmentation DNA was end-repaired, A-tailed and indexed adapters ligated. PCR reaction was performed, using 6 (when starting DNA amount was 200/400ng) or 10 cycles (when starting with 50ng DNA).To optimise enrichment and reduce off target capture, pooled, multiplexed, amplified pre-capture libraries (up to 13 samples per hybridization) were hybridized overnight using 1 µg of total DNA to a custom design of DNA baits complementary to the genomic regions of interest (NimbleGen SeqCap EZ library, Roche, Madison, WI, USA). After hybridisation, unbound capture baits were washed away and the remaining hybridised DNA was PCR amplified (11 cycles). PCR products were purified using AMpure XP beads (Beckman Coulter, Danvers, MA, USA) and quantified using the KAPA Quantification Q-PCR Kit (KAPA Biosystems, Wilmington, MA, USA). Sequencing was performed on a MiSeq (Illumina, San Diego, CA, USA) with 75 bp paired-end reads and v3 chemistry, or NextSeq (Illumina, San Diego, CA, USA) with 75 bp paired-end reads and v2 chemistry, according to the manufacturer’s instructions.

**Data Analysis – MiSeq sequencing**

Primary analysis was performed using MiSeq Reporter Software (v2.5.1; Illumina), generating nucleotide sequences and base quality scores in Fastq format. Resulting sequences were aligned against the human reference genome build GRCh37/Hg19 to generate binary alignment (BAM) and variant call files (VCF). Secondary analysis was performed in-house using Molecular Diagnostics Information Management System to generate QC, variant annotation, data visualisation and a clinical report. In the Molecular Diagnostics Information Management System, reads were deduplicated using Picard (<http://broadinstitute.github.io/picard/>), and metrics generated for each panel region. Oncotator (v1.5.3.0) (https://portals.broadinstitute.org/oncotator) was used to annotate point mutations using a minimum variant allele frequency (VAF) of 5% and a minimum number of 10 variant reads. Variants were annotated for gene names, functional consequence (e.g. Missense), PolyPhen-2 predictions, and cancer-specific annotations from the variant databases including COSMIC, Tumorscape, and published MutSig results. All potential mutations were visualised using Integrative Genomics Viewer (IGV) and two individuals were required to review the mutation report independently.

VCF files from unpaired samples were annotated using Illumina Varinat Studio v3.0, and checked manually on IGV.

**Data Analysis – NextSeq sequencing**

These samples were analysed using an inhouse developed pipeline. For the demultiplexing bcl2fastq (v2.19) was used to isolate reads meant for each sample. The reads were aligned to the reference genome build GRCh37/Hg19 using Burrows-Wheeler Aligner (BWA-MEM), followed by the marking of PCR duplicates and calculation of various quality control (QC) metrics using picard. GATK was used for variant calling using HaploType Caller for tumour only analysis (limit of detection ~10%) and MuTect2 for tumour paired analysis.

VCF files from unpaired samples were annotated using Illumina Varinat Studio v3.0, and checked manually on IGV.

Mutational validation

PCR was run with amplitaq gold, gold buffer and MgCl_2_ following manufacturer’s instructions (Applied Biosystems, Foster City, CA) with primers described in Table S1. Annealing conditions were denaturing step of 95°C 10min, 35 cycles of 95°C 15 sec, 50°C 30 sec and 72°C 1min, and last step 72°C 5min. Sequencing was provided by Eurofins Genomics (Eurofins, Luxembourg) on the ABI3730XL sequencing machine.

**NanoString analysis**

For both codesets, NanostringNorm (<https://CRAN.R-project.org/package=NanoStringNorm>) was used to adjust technical variation (set geometric mean of Positive controls set to 10000), adjust background signal (subtract mean of Negative Controls) and then normalize using geometric mean of 5 housekeeping genes (SF3A1, TBP, PSMC4, MRPL19, and ACTB) (set geometric mean of housekeeping genes to 10000). Finally, normalised data was log2-transformed. As the geometric mean of the housekeeping genes of both codesets was set to the same level, the datasets were merged for each sample by taking the mean log2 value of the 50 probes common to both codesets.

Finally, normalized data was interrogated using custom R scripts. R (v3.2.3) was used for Mann-Whitney tests and generation of box plots, arrow plots, dot plots and heatmaps. Heatmap clustering was based on Pearson correlation and Ward's agglomeration method.

**References**

1. Danecek P, Auton A, Abecasis G et al. The variant call format and VCFtools. Bioinformatics 2011; 27: 2156-2158.

2. Mardis ER, Ding L, Dooling DJ et al. Recurring mutations found by sequencing an acute myeloid leukemia genome. N Engl J Med 2009; 361: 1058-1066.

3. Quinlan AR, Hall IM. BEDTools: a flexible suite of utilities for comparing genomic features. Bioinformatics 2010; 26: 841-842.

4. Wang K, Li M, Hakonarson H. ANNOVAR: functional annotation of genetic variants from high-throughput sequencing data. Nucleic Acids Res 2010; 38: e164.

5. Bamford S, Dawson E, Forbes S et al. The COSMIC (Catalogue of Somatic Mutations in Cancer) database and website. Br J Cancer 2004; 91: 355-358.

6. Wong SQ, Li J, Tan AY et al. Sequence artefacts in a prospective series of formalin-fixed tumours tested for mutations in hotspot regions by massively parallel sequencing. BMC Med Genomics 2014; 7: 23.
