# Supplementary figures and images for: Molecular characterisation of aromatase inhibitor-resistant advanced breast cancer: the phenotypic effect of ESR1 mutations
Source: Br J Cancer. 2018 Dec 19;120(2):247–55. doi: 10.1038/s41416-018-0345-x (PMC6342946; doi:10.1038/s41416-018-0345-x)

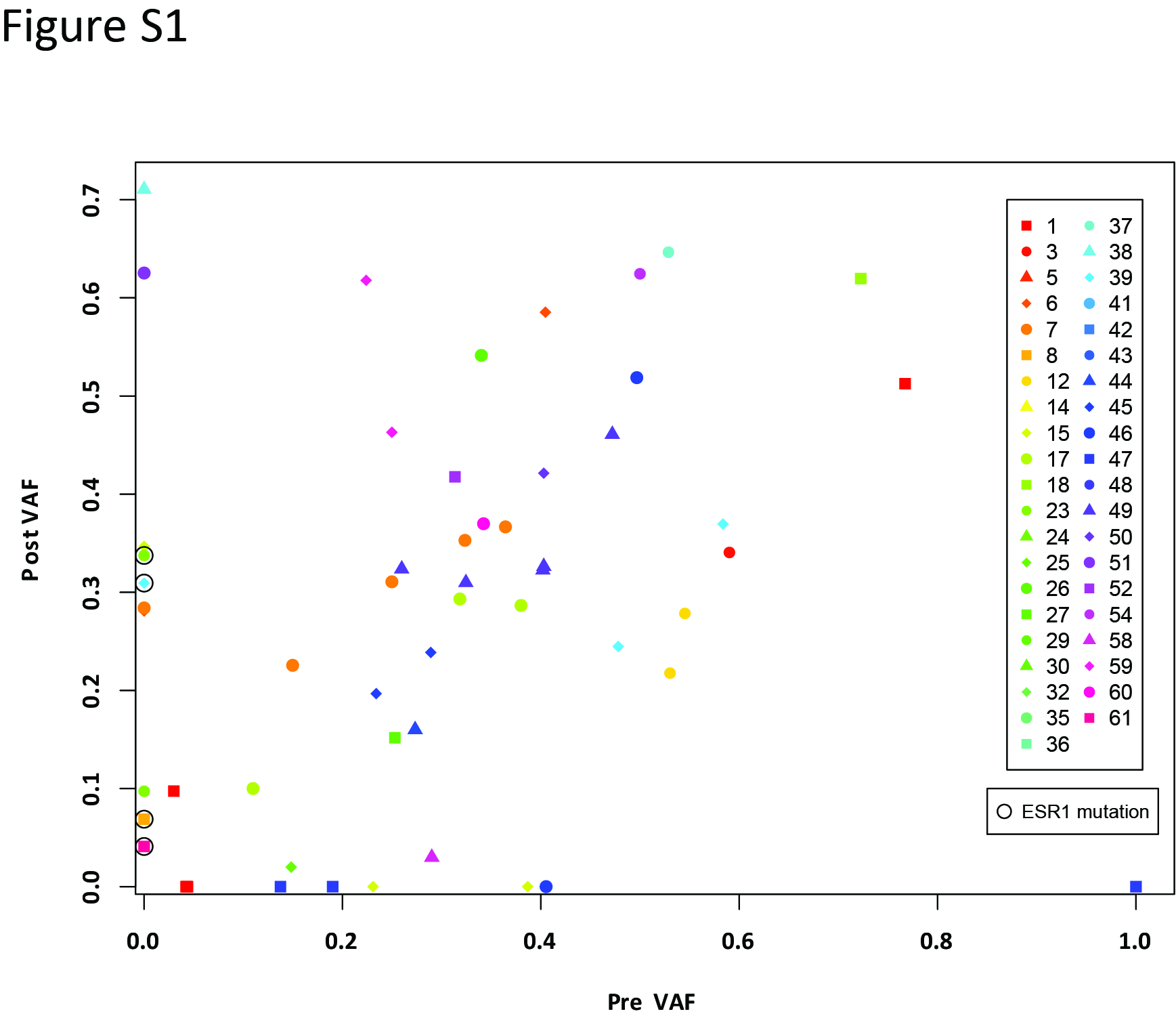

Supplement: Supplementary file 2 — Figure S1 [file 41416_2018_345_MOESM2_ESM.tif]

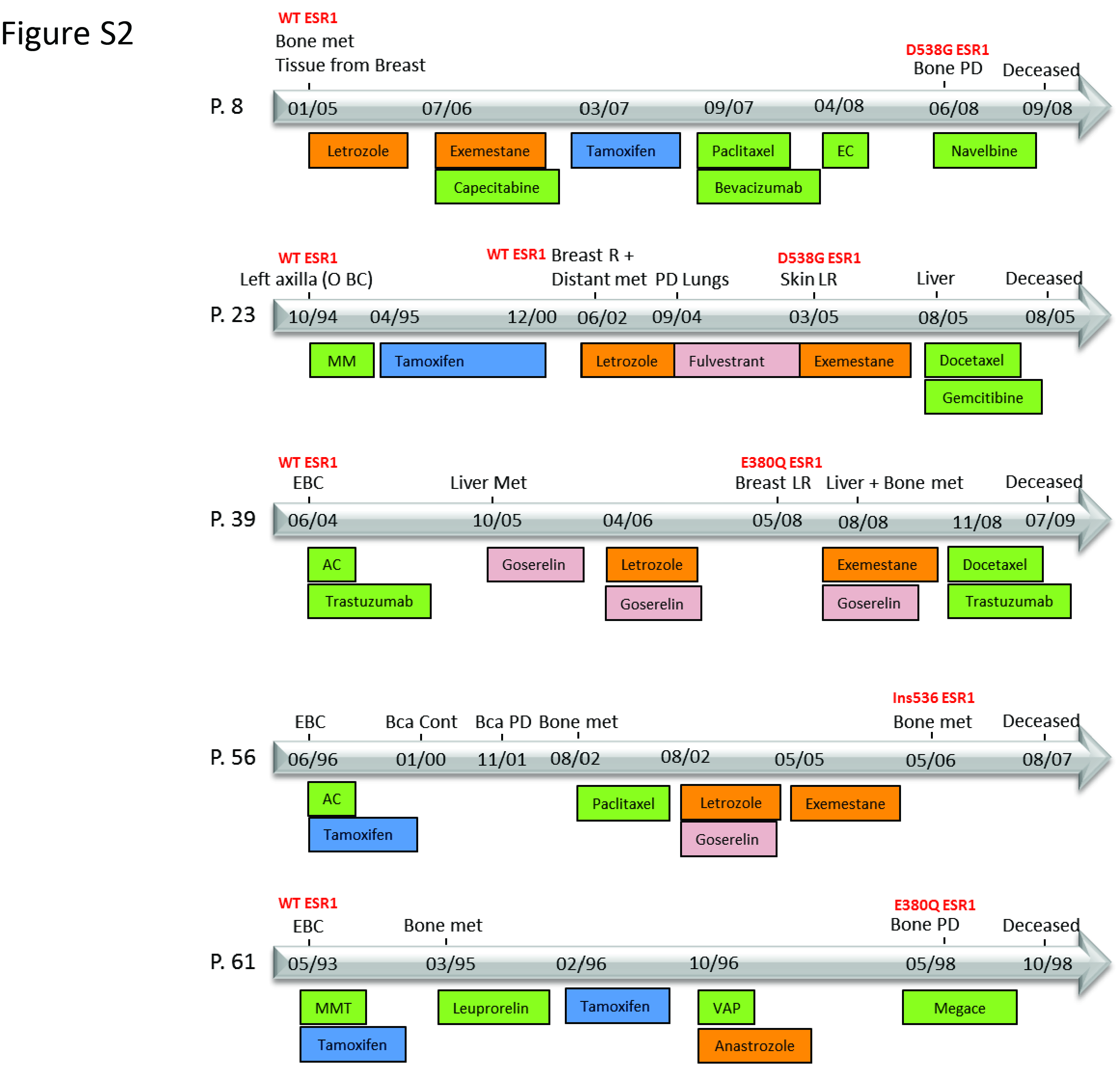

Supplement: Supplementary file 3 — Figure S2 [file 41416_2018_345_MOESM3_ESM.tif]

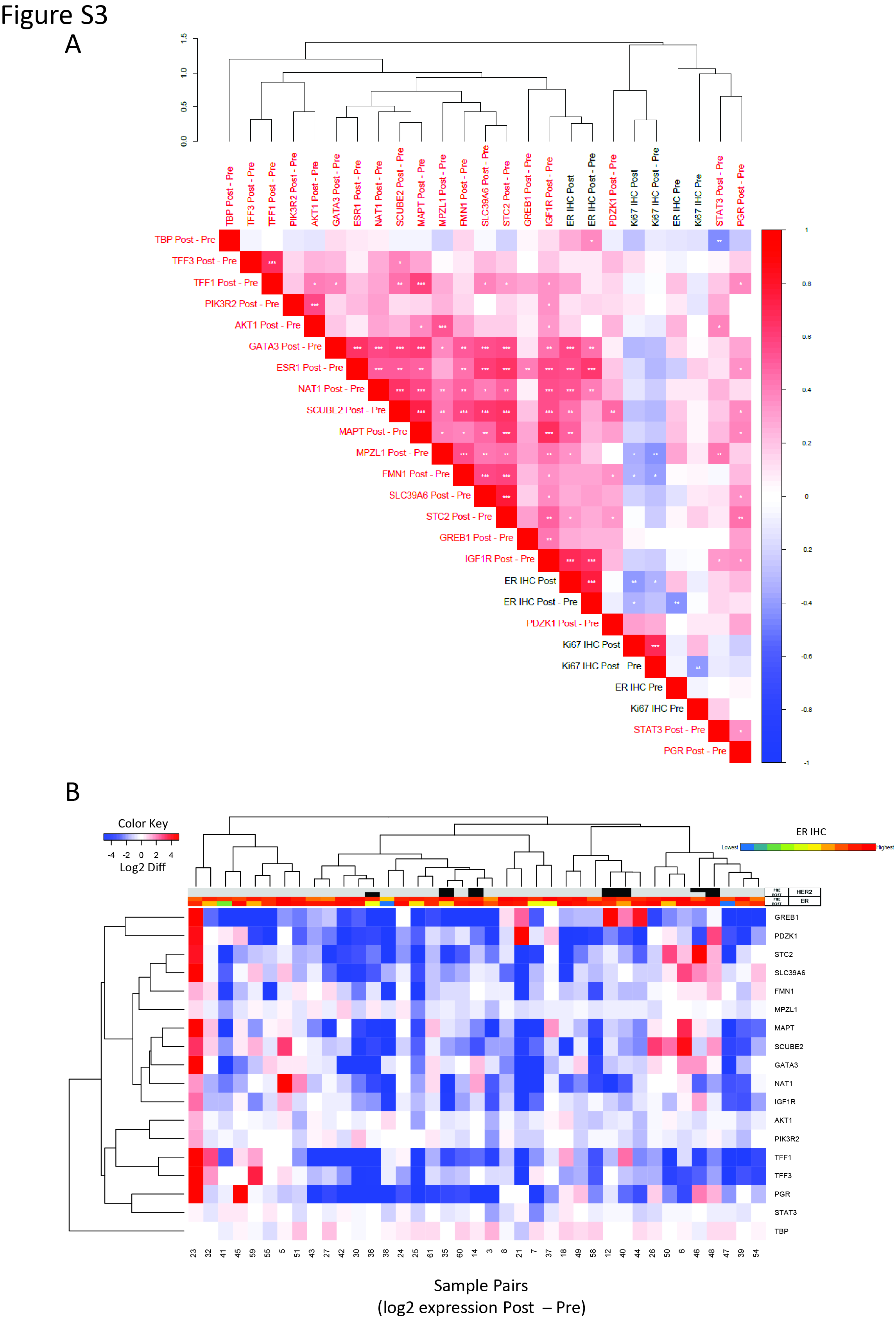

Supplement: Supplementary file 4 — Figure S3 [file 41416_2018_345_MOESM4_ESM.tif]

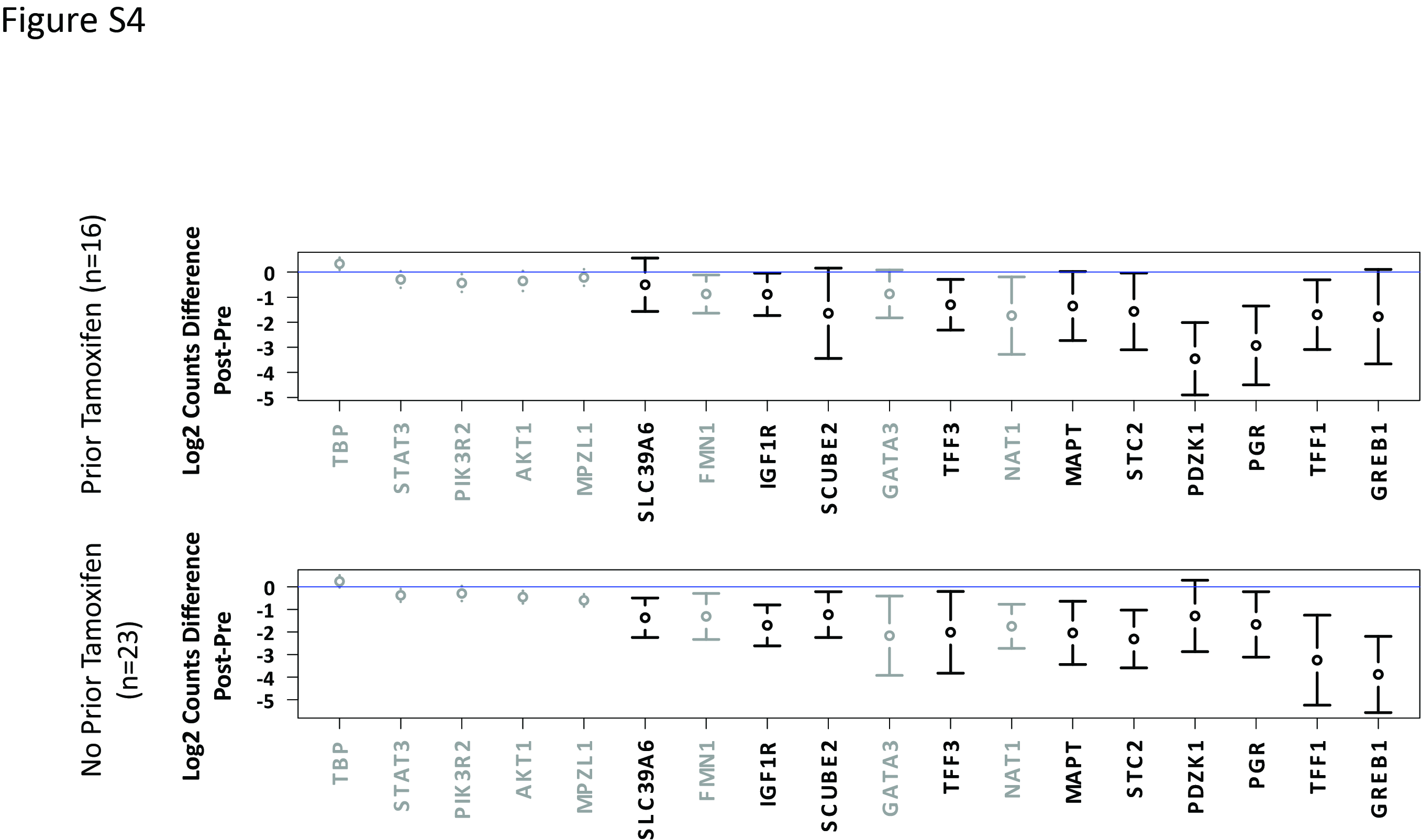

Supplement: Supplementary file 5 — Figure S4 [file 41416_2018_345_MOESM5_ESM.tif]

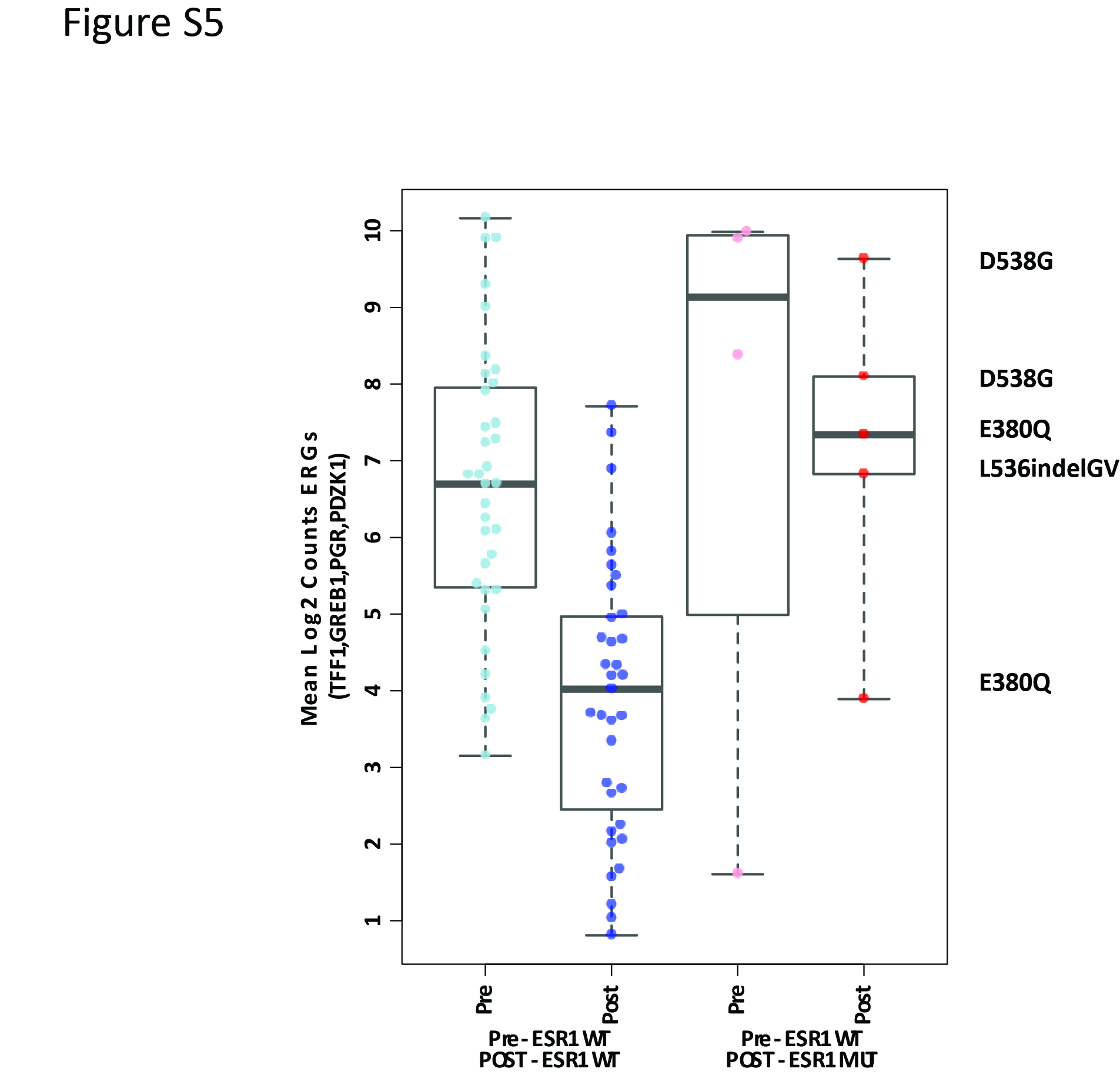

Supplement: Supplementary file 6 — Figure S5 [file 41416_2018_345_MOESM6_ESM.tif]

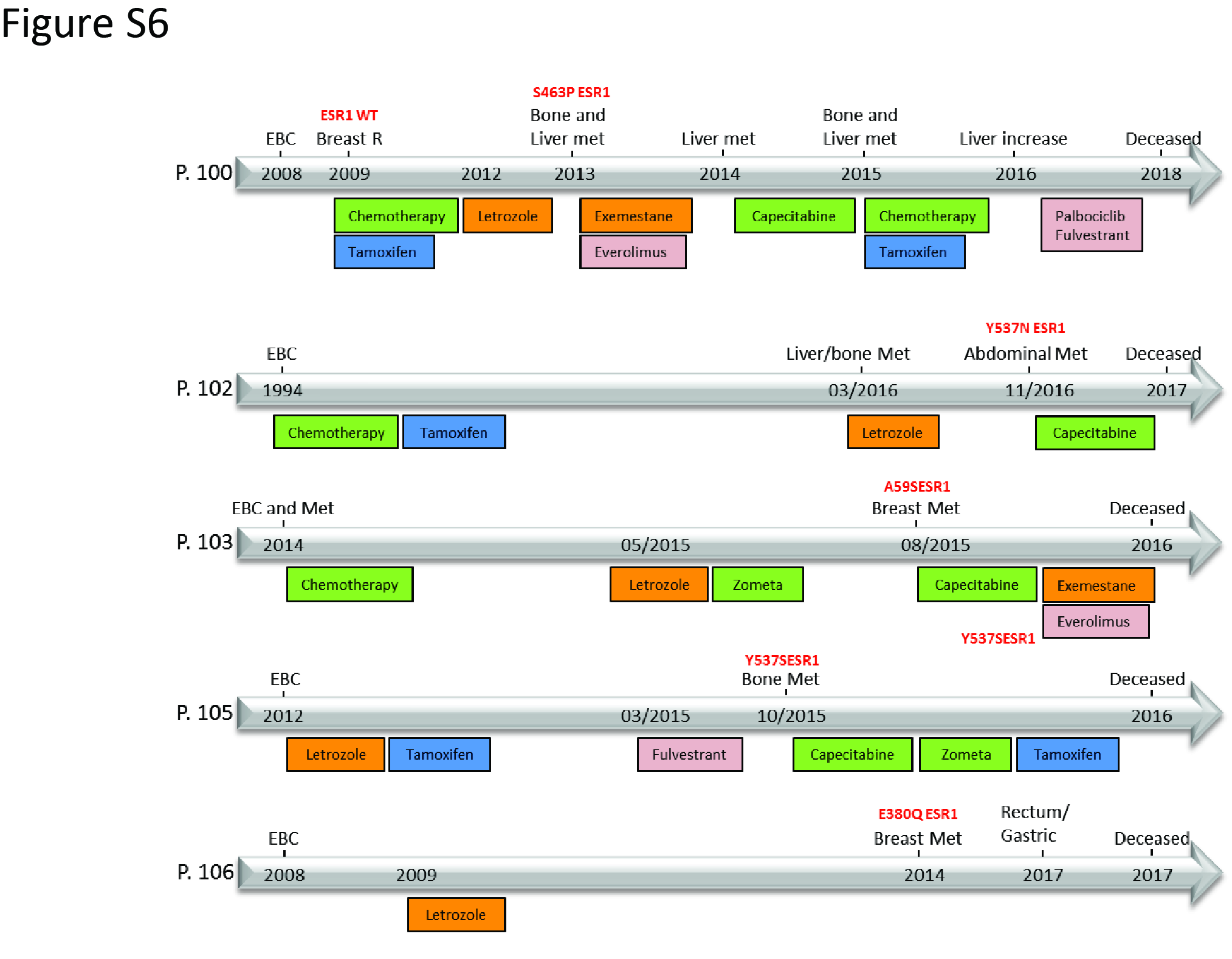

Supplement: Supplementary file 7 — Figure S6 [file 41416_2018_345_MOESM7_ESM.tif]

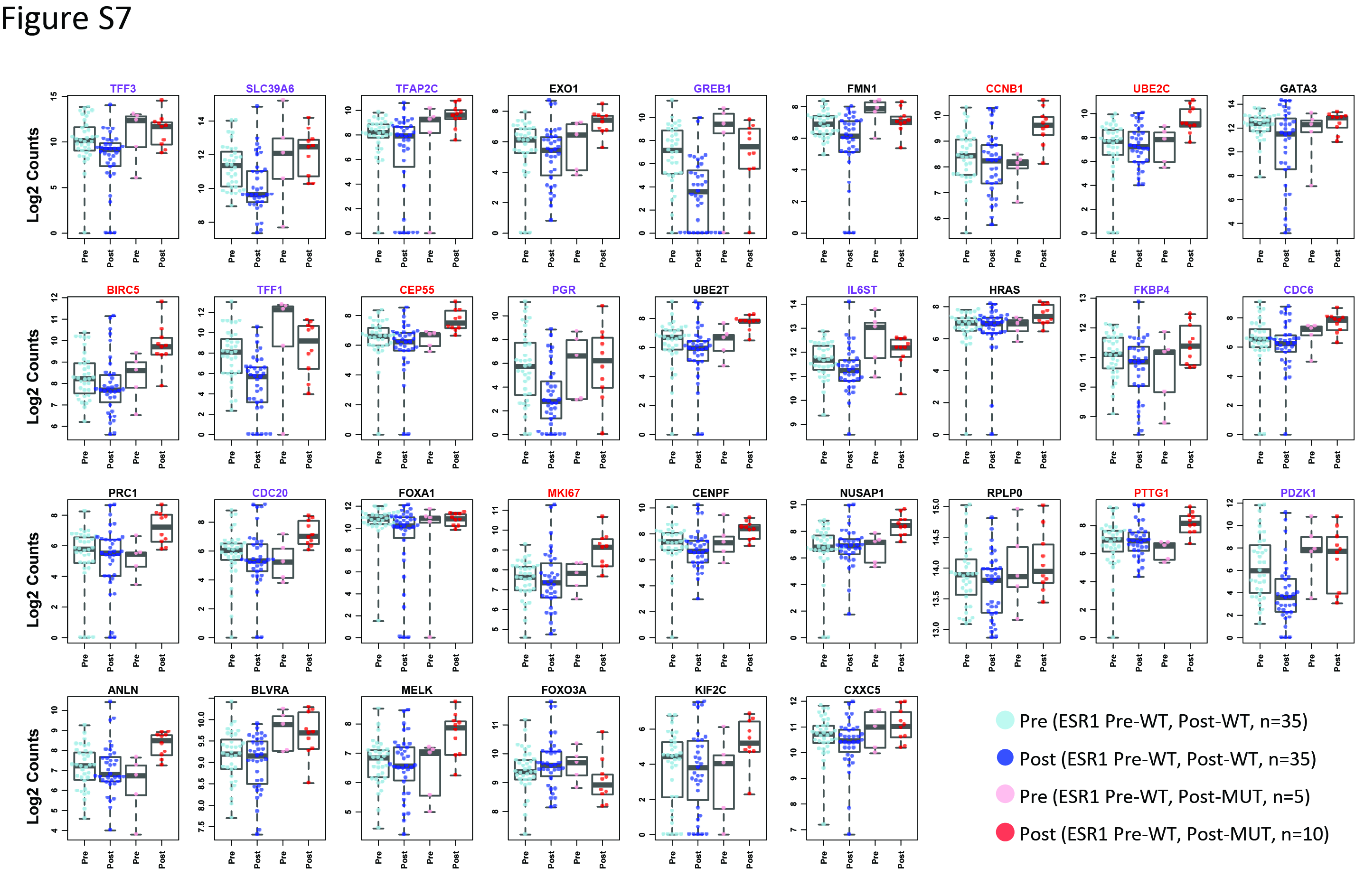

Supplement: Supplementary file 8 — Figure S7 [file 41416_2018_345_MOESM8_ESM.tif]

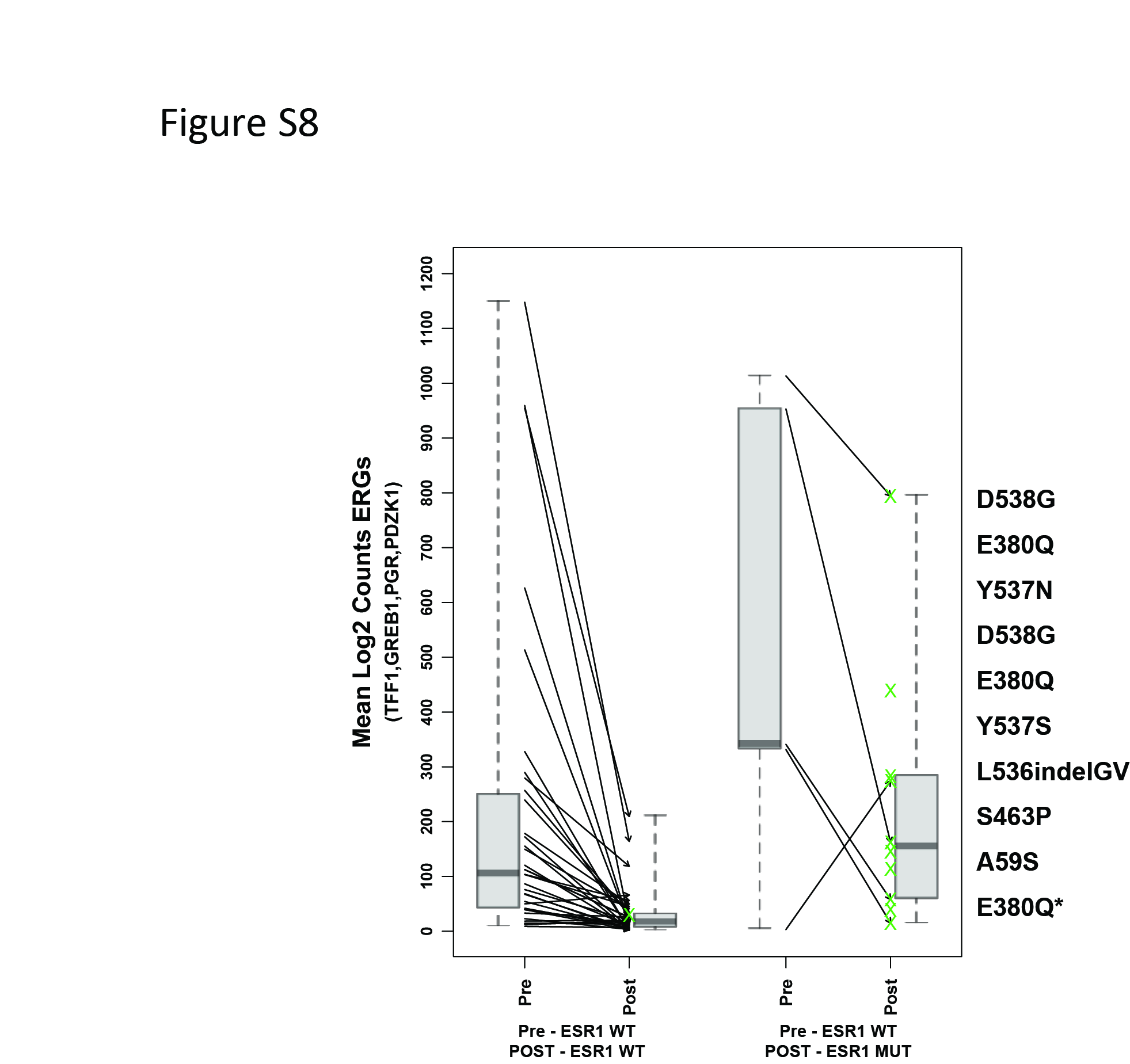

Supplement: Supplementary file 9 — Figure S8 [file 41416_2018_345_MOESM9_ESM.tif]
